# Patient satisfaction of radiofrequency ablation for symptomatic benign solid thyroid nodules: our experience for 2-year follow up

**DOI:** 10.1186/s12885-019-5338-5

**Published:** 2019-02-13

**Authors:** Yang Guang, Wen He, Yukun Luo, Hongxia Zhang, Yukang Zhang, Bin Ning, Tengfei Yu

**Affiliations:** 1 Department of Ultrasound, Beijing Tian Tan Hospital, Capital Medical University, No. 119 West Road of South 4th Ring Road, Fengtai District, Beijing, 100160 China; 2 Department of Ultrasound, Chinese PLA General Hospital, No.28 Fuxing Road, Haidian District, Beijing, 100853 China

**Keywords:** Radiofrequency ablation (RFA), Thyroid nodules, Ultrasound

## Abstract

**Background:**

The patient satisfaction of symptoms improvement and disease factors that may affect long-term treatment efficacy of radiofrequency ablation (RFA) for non-functioning solid benign thyroid nodules (TNs) over a 2-year follow up study was investigated.

**Methods:**

This retrospective study evaluated 194 non-functioning solid benign TNs of 103 patients. The TNs were categorized as small (≤5 ml), medium (5.1 to 13 ml), intermediate (13.1 to 30 ml) and large (over 30 ml) according to the initial volume of TNs before ablation. Clinical evaluation and contrast-enhanced ultrasound (CEUS) were carried out before ablation and the follow up at 1, 3, 6 months and every 6 months after ablation. All patients were asked to assess the cosmetic score (1–4 scores) and symptom score (0–10 scores) before ablation and every follow up after ablation.

**Results:**

All patients underwent RFA without any major complications. The mean treatment sessions were 1.5 ± 0.6. 98 nodules required a single session (98/194, 50.5%), 87 required two sessions (87/194, 44.9%), 9 required three sessions (9/194, 4.6%). The average follow up months were 16.3 ± 5.6 (range, 6–24 months) and no nodule regrew in our study. After RFA treatment, the TNs volume significantly decreased (*P* < 0.001). The small group of nodules shrunk larger compared to the medium, intermediate and large groups (*P* < 0.001). Cosmetic signs and pressure symptoms were significantly improved, particularly in the intermediate and large groups (*P* < 0.05).

**Conclusions:**

RFA is effective for treating non-functioning solid benign TNs and controlling clinical symptoms with a low complication rate during 2 years follow up. The reduction rate was related to the initial volume of nodules. Patients were satisfied with cosmetic signs and pressure symptoms improvement, particularly in the intermediate and large groups. However, multiple RFA treatments should be used in larger nodules to achieve the desired clinical outcomes.

## Background

The incidence of thyroid nodules (TNs) is fairly high, with the development of medical imaging technologies the presence of thyroid nodule increases up to 40–50% [1, 2]. Some studies have shown that most TNs are benign, however, about 20% of TNs grow over time [3]. The compression of the surrounding structures of TNs often leads to discomfort. Large TNs may also lead to life-threatening conditions such as acute respiratory arrest. Research demonstrated that TNs diagnosed by ultrasound guided core needle biopsy (CNB) as benign nodules may be diagnosed as malignant nodules by postoperative pathology, of which the probability is 6% [4]. Therefore, symptomatic benign TNs and suspicious malignant TNs should be actively treated [5, 6]. Traditional surgery can remove the symptomatic thyroid nodule and reduce the possibility for malignancy change. Although the surgical resection is effective, it usually has some risks [7, 8]. Due to several surgical drawbacks and ineligibility, minimally invasive non-surgical technologies have been required in treating TNs [9].

Radiofrequency ablation (RFA) has been successfully applied in treating liver tumors [10]. Because of the advantages of safety, efficacy, no incision and good cosmetic result, RFA has been applied in treating benign TNs and malignant TNs that are not suitable for surgery [11–13]. Although studies have shown that nodules generally continue to shrink while obstructive symptoms continue to improve over time, there were still limitations on small samples of patients and short-term follow up in previous initial studies, or studies in previous reviews [13–16]. However, despite being a promising form of ablation, the medium to the long-term efficacy following RFA treatment for symptomatic benign solid TNs remains unclear. It is uncertain whether these results can continue beyond 2 years. Also, to ensure treatment success in the long-term, it would be important to identify patient satisfaction of symptoms improvement or disease factors that may affect the long-term treatment efficacy. With these issues, therefore, the purpose of this study was to access the effectiveness and clinical outcomes of RFA for non-functioning solid benign TNs over 2-year follow up on a large sample of patients. To the best of our knowledge, it is the first study to evaluate both treatment effect and patients satisfaction for the treatment of benign solid TNs. In this study TNs were categorized according to the baseline volume. The treatment effect, the volume reduction of TNs, RFA sessions, complications and patient satisfaction were evaluated, which may provide reference for clinical work in the future.

## Methods

All patients were required to sign informed consents before contrast-enhanced ultrasound (CEUS), CNB and RFA. This retrospective study was approved by the Beijing Tian Tan Hospital Ethics Committee and the Chinese PLA General Hospital Ethics Committee. From June 2014 to July 2015, 412 TNs of 270 patients received RFA for treating TNs in Beijing Tian Tan Hospital and Chinese PLA General Hospital. Patients enrolled in this study were based on the following criteria: (a) pathological diagnosis was benign by ultrasound guided CNB of thyroid nodule; (b) TNs with solid portion over 80%; (c) thyroid stimulating hormone (TSH), anti-thyroid antibodies and free T3/T4 within the normal range; (d) patients with normal electrocardiogram (ECG) and chest X-ray; (e) patients reported foreign body sensation, compression symptom and local uplift affection due to large volume of nodules, and worries of nodules growing rapidly or malignant lesions; (f) refusal or ineligible for surgery; (g) patients voluntarily receive RFA after being informed of the treatment features of RFA. Exclusion criteria were: (a) the distance from TNs to trachea-esophageal groove is less than 5 mm; (b) pathological diagnosis was malignant by ultrasound guided CNB; (c) patients with contra-lateral vocal cord paralysis; (d) patients diagnosed with serious disease that can’t tolerate RFA; (e) Pregnancy. Finally, 103 patients with a total of 194 TNs (36 males, 67 females) were enrolled in this study and more than 2 years follow up was conducted. At the enrollment, all patients were asked to assess the cosmetic score (1–4 scores) (score 1, no palpable mass; score 2, no cosmetic problem but a palpable mass; score 3, cosmetic problem on swallowing only; and score 4, a readily observable cosmetic problem) and rate symptom score (0–10 scores) on a 10 cm visual analog scale [17, 18].

The equation of volume of thyroid nodule was V = abcπ/6, (a, b, c: transverse diameter, vertical diameter, anteroposterior diameter of nodules) [17]. TNs were categorized as small (≤5 ml), medium (5.1 to 13 ml), intermediate (13.1 to 30 ml) and large (over 30 ml) [19]. We performed CEUS to observe the blood supply of TNs before and after ablation as well as during the follow up period. Contrast agent was SonoVue (2.4 ml, MI: 0.19–0.24) followed by saline flush (5 ml). RFA was performed on Olympus Surgical Technologies (CelonLabPOWER, Europe, Germany, Hamburg), 9-gauge/15-gauge bipolar radiofrequency applicator with 9 mm/15 mm active tip. The ablation procedure referred to our previous study [20]: Patients were supine with the neck extended during the procedure. An iv line was introduced via the antecubital vein. Before RFA, the relationship between thyroid nodules and cervical critical structures such as trachea, vessels, esophagus and recurrent laryngeal nerves was carefully evaluated by the operator in order to design the best insertion way. Local anesthesia with 1% lidocaine was injected at the subcutaneous puncture site and the periphery of thyroid nodules. If the distance between the thyroid nodules and critical cervical structures (including trachea, cervical artery, jugular vein, esophagus and recurrent laryngeal nerve) was less than 5 mm, saline was injected using another needle (23 gauge) to form at least 1 cm distance between the nodule and the critical structure in order to prevent significant complications (i.e., perinodular hematoma, nerve injury, local infection, skin burn or hypothyroidism etc.). RFA was performed using the “moving-shot technique” [11]: each nodule was divided into multiple small conceptual units and was treated in a unit-by-unit manner by moving the RFA electrode tip. The tip of the electrode was initially positioned in the deepest portion of the nodule, which enabled easy monitoring of the tip without disturbance caused by hyperechoic cloud from the gas generated during the ablation procedures. The RFA power was set up to 3 W. The power was increased to 5 W–8 W if there was no hyperechoic area at the electrode tip in 5–10 s. Patients were asked to stay for 1–2 h in the hospital to observe any possible complication after ablation.

After ablation, patients were followed up at 1, 3, 6, 12 and every 6 months thereafter. Symptom and cosmetic scores and complications of RFA were evaluated at each follow up. CEUS was used to evaluate for recurrence and detect the volume of ablation. Volume reduction ratio (VRR) was calculated by the following equation: VRR = [Initial Volume (ml) – Final Volume (ml)]/Initial Volume (ml) × 100% [17]. Regrowth was defined as the nodule volume increased 50% compared to the previous examination. Additional ablation was based on the following criteria: (a) the volume of nodules didn’t decrease than the previous examination; (b) CEUS showed the thickness of the active tissue in the nodules exceeded 10 mm; (c) patients reported foreign body sensation, compression symptom and local uplift affection didn’t relief. After multiple ablations, the follow up period for each turn of ablation was every 6 months and the follow-up time was calculated from the first ablation.

SPSS 17.0 software was used to perform statistical analysis. Changes in VRR, symptom and cosmetic scores between every two follow up periods were compared by Wilcoxon signed rank tests. *P* value less than 0.05 indicated there was significant statistical difference.

## Results

RFA was applied in 194 TNs of 103 patients in this study, 36 male and 67 female. The average age was 47.6 ± 11.3 years (range, 19–82 years). TNs were situated at left lobe, right lobe and isthmus in 81, 103 and 10 cases, respectively. The mean initial volume of TNs was 21.2 ± 19.7 ml (range, 1.1–91.1 ml). Table 1 summarized the characteristic parameters of the TNs of the patients.

**Table 1:** Main characteristics of the study population and clinical data of RFA treated TNs at baseline

Parameter	Characteristics	Result
Sex of patients (*n* = 103)	M/F	36/67^a^
Age of patients (n = 103)		47.6 ± 11.3 (19–82)^b^
Location of nodules (*n* = 194)	L	81 (41.7)^c^
	R	103 (53.1)^c^
	I	10 (5.2)^c^
Initial volume of nodules (n = 194) (ml)		21.2 ± 19.7 (1.1–91.1)^b^
	≤5 ml (*n* = 38)	3.7 ± 1.1 (1.1–4.9)^b^
	5.1–13 ml (*n* = 86)	7.8 ± 2.6 (5.1–12.8)^b^
	13.1–30 ml (*n* = 43)	19.8 ± 4.3 (13.7–28.5)^b^
	>30 ml (*n* = 27)	48.6 ± 16.4 (31.5–91.1)^b^

*M* male, *F* female*, L* left, *R* right, *I* isthmus

^a^Number of patients

^b^Means ± standard deviations, with range in parentheses

^c^Number of thyroid nodules, with percentages in parentheses

It was possible to successfully perform the RFA in all cases as planned. Power of 3 W was used in 21 nodules; 5 W – 6 W was used in 119 nodules; 7 W – 8 W was used in 54 nodules. No immediate or later major complications occurred during and after RFA. Mild to moderate local pain occurred during RFA in 12 patients (12/103, 11.6%) and lasted for 1 to 3 days and alleviated with analgesics. The average sessions of RFA treatment were 1.5 ± 0.6. 98 nodules required a single session (98/194, 50.5%); 87 required two sessions (87/194, 44.9%); 9 required three sessions (9/194, 4.6%).

**Fig. 1:**
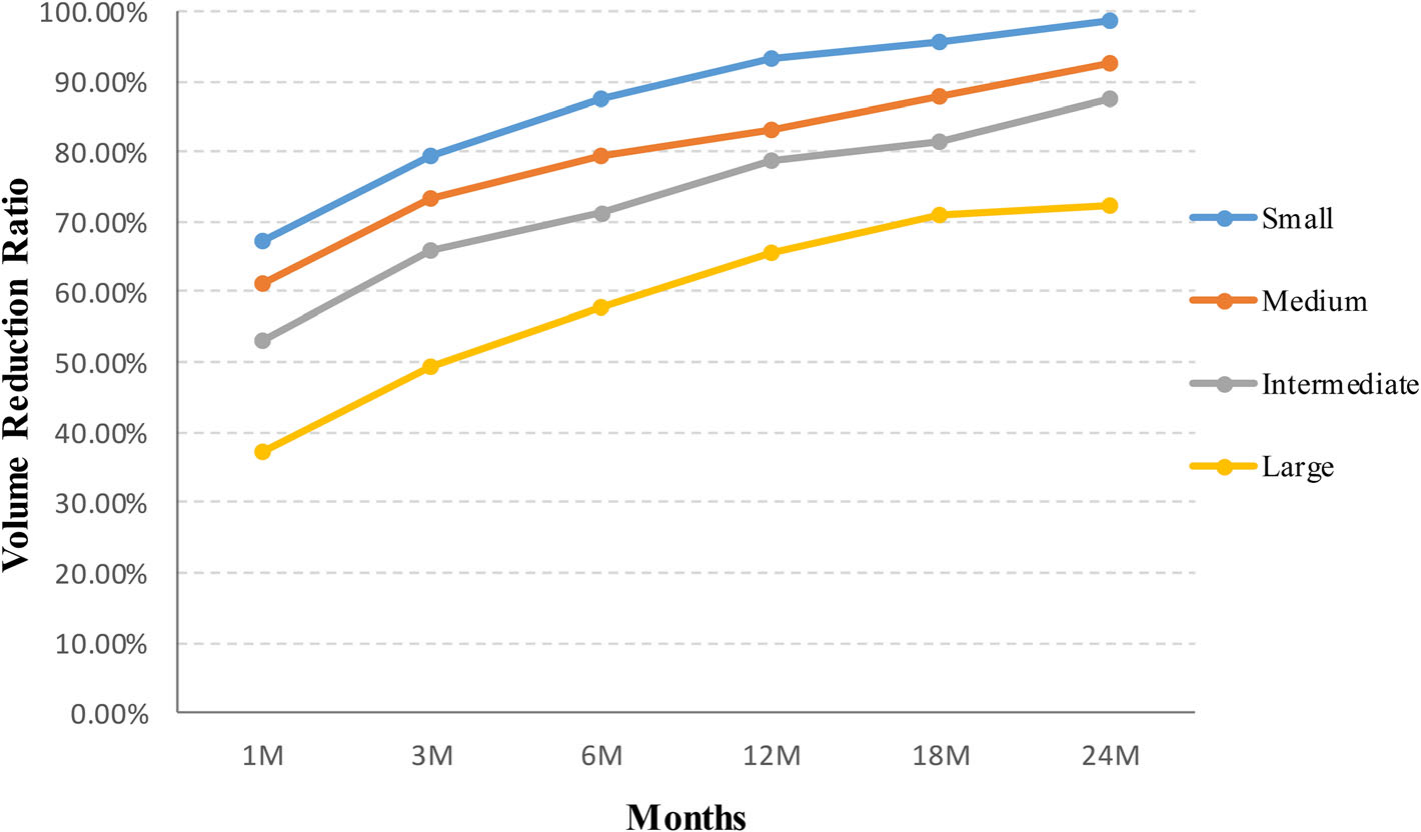
Changes in VRR of the four groups of 194 non-functioning solid benign thyroid nodules at each follow-up

**Fig. 2:**
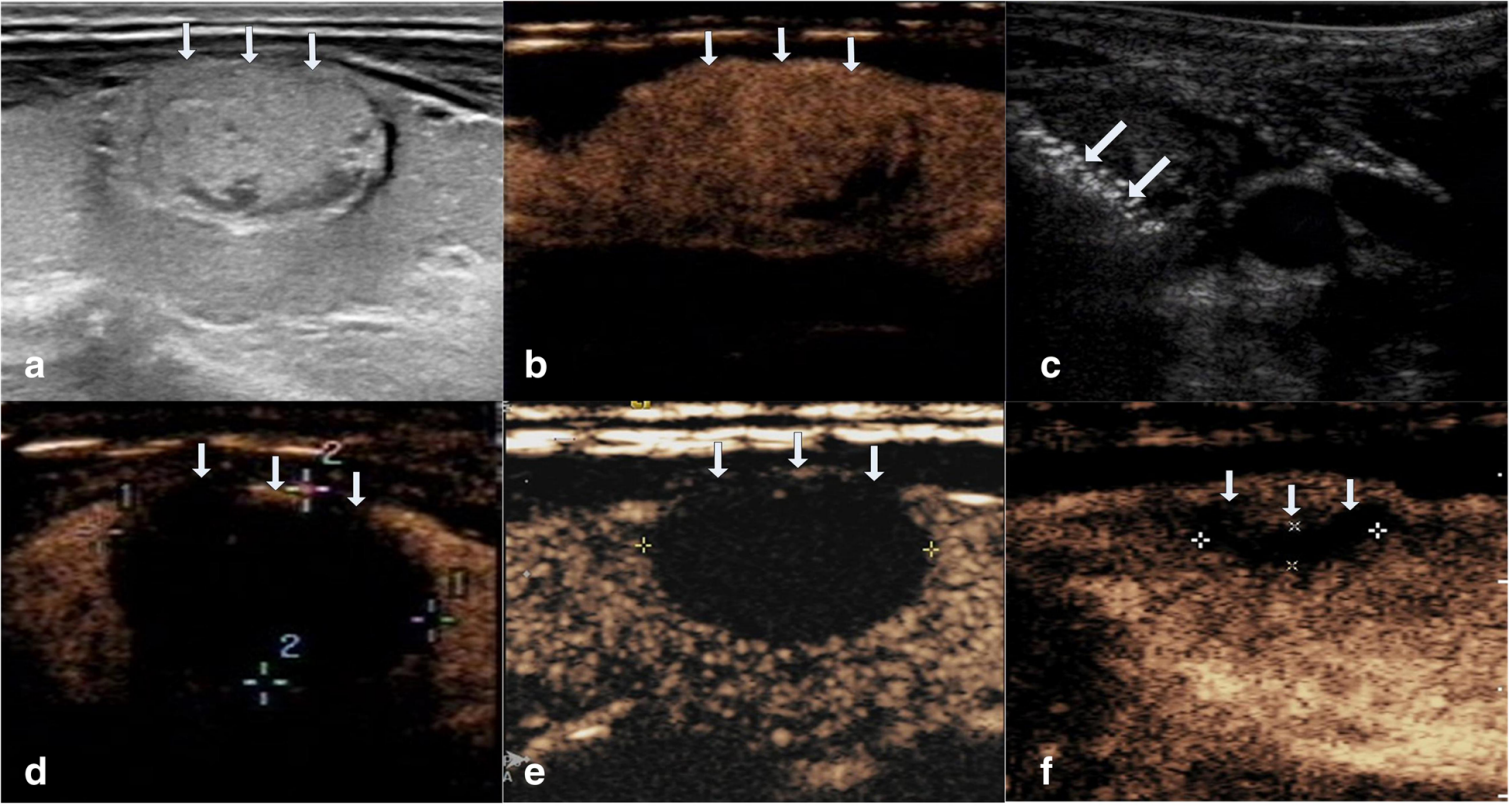
Images of RFA treatment and follow-up of a thyroid nodule at the left lobe in small group. **a** Conventional ultrasound image shows a solid thyroid nodule. **b** CEUS performed before RFA shows an irregular enhancement in nodule (white arrows). **c** During RFA, ultrasound monitoring of the procedure shows gas formation in the nodule which covered by a hyperechoic ablation area (white arrows). **d** CEUS performed immediately after RFA shows a complete lack of enhancement in the treated area (white arrows). **e** One month after RFA, CEUS shows the treated area shrunk and that complete lack of enhancement (white arrows). **f** 1-year after RFA, CEUS shows complete lack of enhancement in the treated area, remained as a small scarlike lesion (white arrows)

**Fig. 3:**
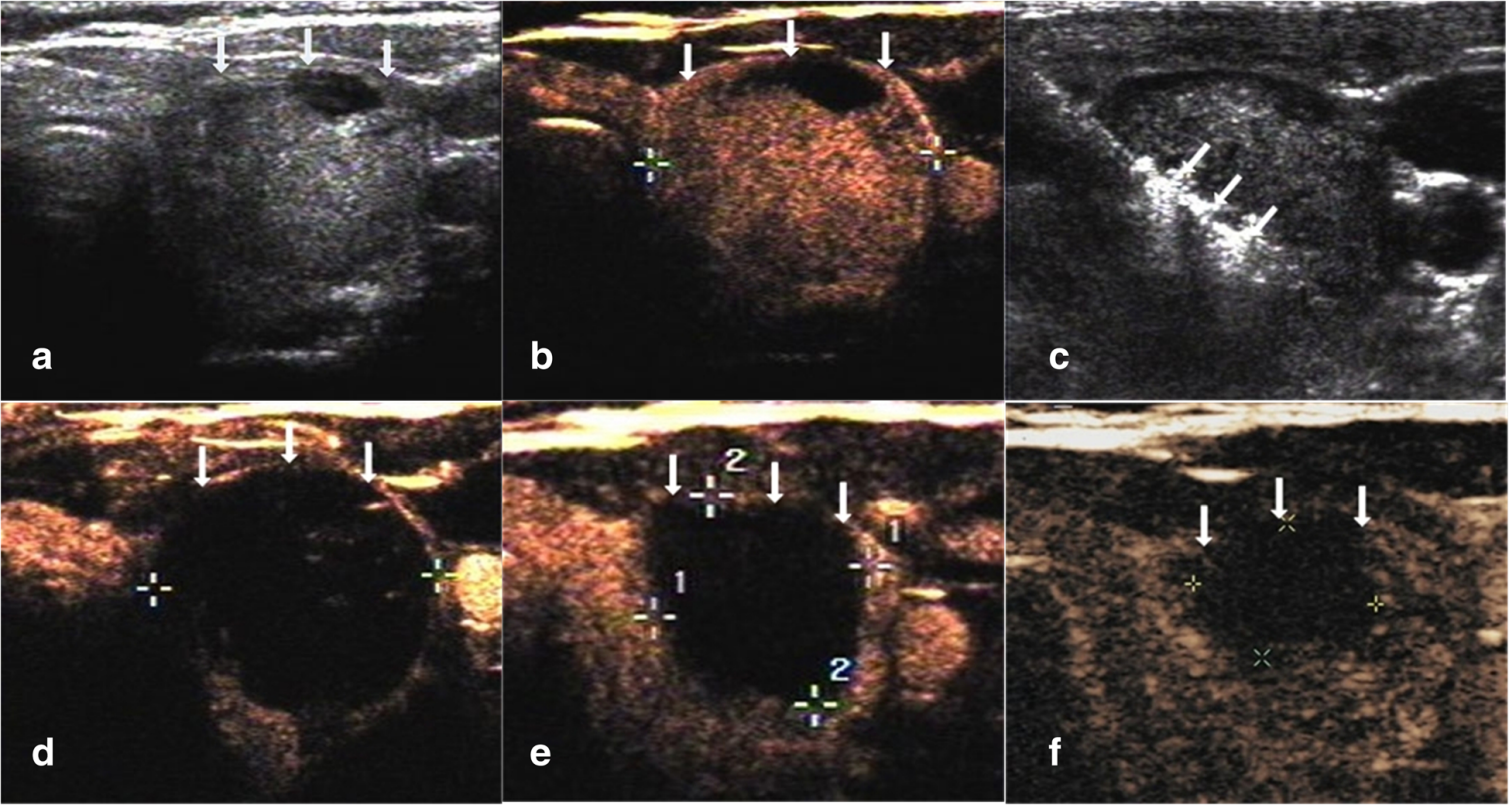
Images of RFA treatment and follow-up of a thyroid nodule at left lobe in medium group. **a** Conventional ultrasound image shows a thyroid nodule with solid portion over 80%. **b** CEUS performed before RFA shows an irregular enhancement in nodule with a regular hyperechoic ring around the nodule (white arrows). **c** During RFA, ultrasound monitoring of the procedure shows gas formation in the nodule which covered by a hyperechoic ablation area (white arrows). **d** CEUS performed immediately after RFA shows a complete lack of enhancement in the treated area (white arrows). **e** One month after RFA, CEUS shows the treated area shrunk and that complete lack of enhancement (white arrows). **f** 1-year after RFA, CEUS shows complete lack of enhancement in the treated area, remained as a small scarlike lesion (white arrows)

**Fig. 4:**
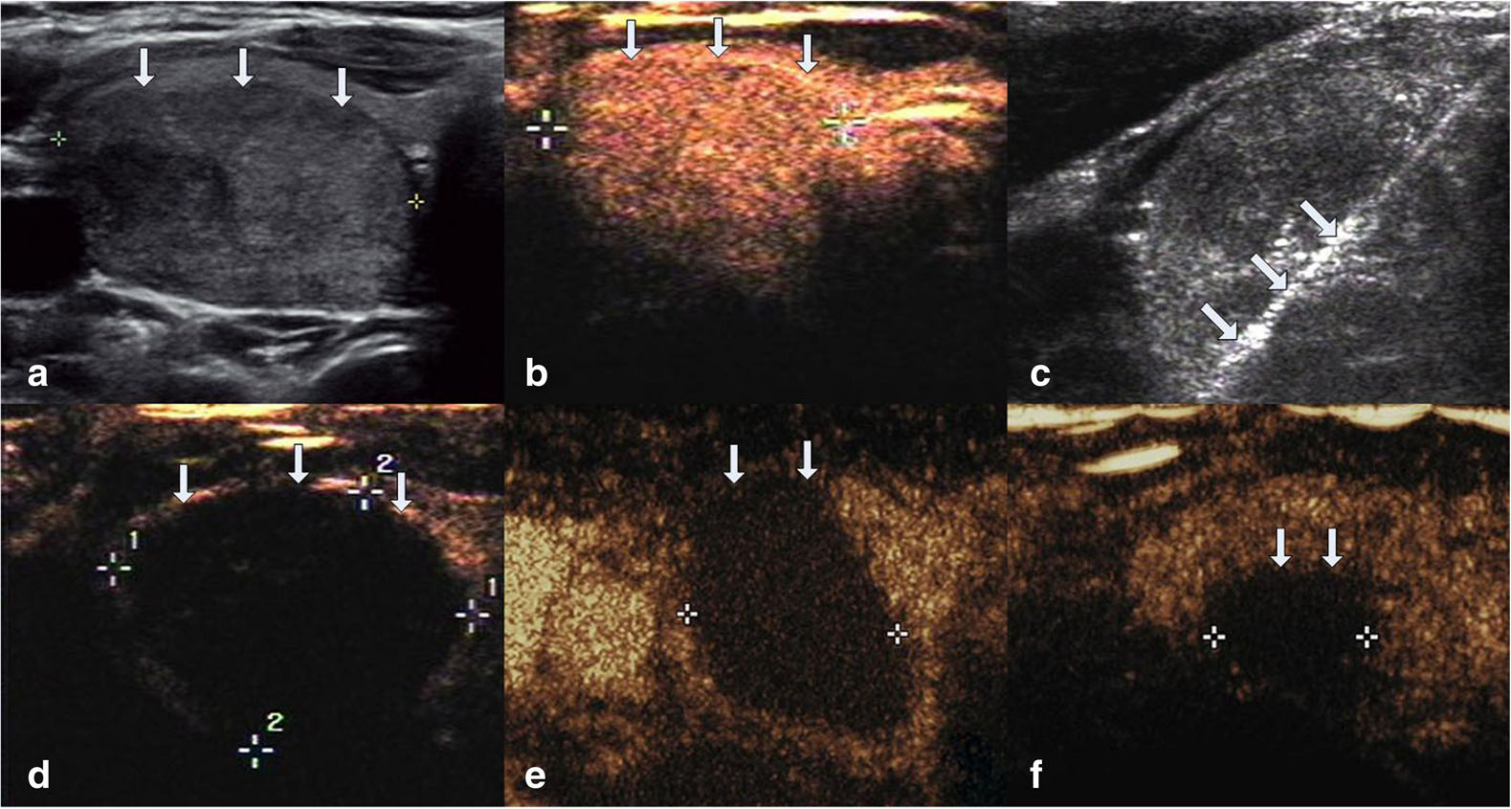
Images of RFA treatment and follow-up of a thyroid nodule at right lobe in intermediate group. **a** Conventional ultrasound image shows a solid thyroid nodule. **b** CEUS performed before RFA shows an irregular enhancement in nodule (white arrows). **c** During RFA, ultrasound monitoring of the procedure shows gas formation in the nodule which covered by a hyperechoic ablation area (white arrows). **d** CEUS performed immediately after RFA shows a complete lack of enhancement in the treated area (white arrows). **e** Three months after RFA, CEUS shows the treated area shrunk and that complete lack of enhancement (white arrows). **f** 1.5-year after RFA, CEUS shows complete lack of enhancement in the treated area, remained as a small scarlike lesion (white arrows)

**Fig. 5:**
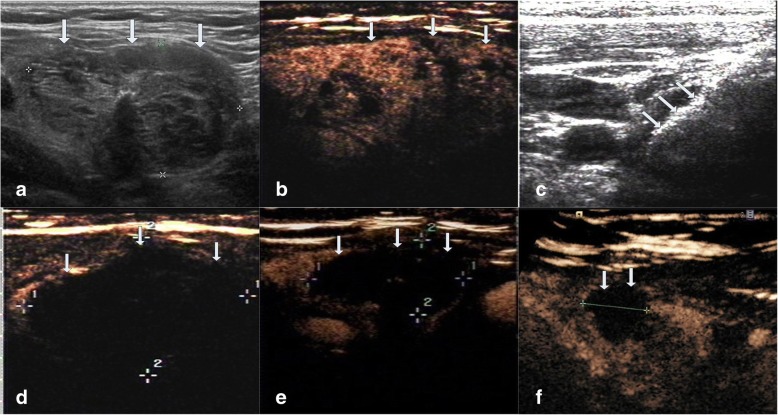
Images of RFA treatment and follow-up of a thyroid nodule at right lobe in large group. **a** Conventional ultrasound image shows a thyroid nodule with solid portion over 80%. **b** CEUS performed before RFA shows an irregular enhancement in nodule (white arrows). **c** During RFA, ultrasound monitoring of the procedure shows gas formation in the nodule which covered by a hyperechoic ablation area (white arrows). **d** CEUS performed immediately after RFA shows a complete lack of enhancement in the treated area (white arrows). **e** Six months after RFA, CEUS shows the treated area shrunk and that complete lack of enhancement (white arrows). **f** 2-year after RFA, CEUS shows complete lack of enhancement in the treated area, remained as a small scarlike lesion (white arrows)

The average follow up months were 16.3 ± 5.6 (range, 6–24 months) in this study. The VRR at each follow up after RFA in the four groups of nodules are shown in Table 2 and Fig. 1. The volume of TNs decreased significantly (*P* < 0.001). During the follow up period, CEUS showed no nodule regrew (regrowth is defined as the nodule volume increased 50% more than the previous examination). 39 nodules completely disappeared (39/194, 20.1%). 27 nodules were in small group (27/38, 71.1%) and 12 nodules were in medium group (12/86, 13.9%) at the last follow up visit. Figures 2, 3, 4, 5 showed representative case of RFA treatment and follow up of thyroid nodules in each group (small, medium, intermediate and large).

**Table 2:** Changes in VRR of TNs after RFA treatment at each follow-up

	Small ≤ 5 ml (*n* = 38)		Medium 5.1–13 ml (*n* = 86)		Intermediate 13.1–30 ml (*n* = 43)		Large >30 ml (*n* = 27)	
	Mean ± SD	Range	Mean ± SD	Range	Mean ± SD	Range	Mean ± SD	Range
1 month	67.2 ± 10.7	37.2–86.3	61.2 ± 11.1	24.5–80.3	53.1 ± 15.7	15.6–79.3	37.1 ± 16.2	12.3–54.3
3 months	79.5 ± 9.6^a^	43.3–100	73.3 ± 10.8^a^	35.2–91.2	65.9 ± 13.1^a^	28.1–84.5	49.3 ± 15.6^a^	27.3–65.3
6 months	87.6 ± 8.2^a^	54.7–100	79.4 ± 9.4^a^	46.7–100	71.4 ± 12.6^a^	41.5–89.3	57.8 ± 14.9^a^	38.2–74.7
12 months	93.3 ± 5.9^a^	69.4–100	83.1 ± 7.2^a^	63.2–100	78.6 ± 11.5^a^	56.3–90.6	65.6 ± 13.4^a^	47.2–81.3
18 months	95.5 ± 3.7^a^	76.2–100	87.8 ± 5.3^a^	73.6–100	81.4 ± 9.3^a^	59.7–91.7	70.8 ± 11.1^a^	57.1–86.4
24 months	98.7 ± 1.3^a^	85.6–100	92.4 ± 3.2^a^	78.2–100	87.5 ± 4.6^a^	61.8–93.6	72.3 ± 10.3^a^	60.7–89.7

*VRR* volume reduction ratio, *TNs* thyroid nodules, *RFA* radiofrequency ablation,

^a^ < .001

Patients achieved a significant decrease in the cosmetic and symptom scores between baseline and the last follow up (Table 3). Symptom score significantly improved in the intermediate and large groups (*P* < 0.05), while no improvement in the small and medium groups. Cosmetic score significantly improved in all groups (*P* < 0.05).

**Table 3:** Changes in cosmetic score and symptom score of TNs at baseline and the last follow-up

	Small 5 ml (n = 38)		Medium 5.1–13 ml (n = 86)		Intermediate 13.1–30 ml (n = 43)		Large >30 ml (n = 27)	
	Cosmetic score	Symptom score	Cosmetic score	Symptom score	Cosmetic score	Symptom score	Cosmetic score	Symptom score
Baseline^a^	1.5 ± 0.5	0 ± 0	2.0 ± 0.6	0 ± 0	2.7 ± 0.7	3.8 ± 2.1	3.2 ± 0.7	7.1 ± 1.8
Last follow-up visit^b^	1.0 ± 0^c^	0 ± 0	1.2 ± 0.4^c^	0 ± 0	1.5 ± 0.4^c^	0.7 ± 0.5^c^	1.7 ± 0.5^c^	0.9 ± 0.6^c^

*TNs* thyroid nodules

^a^The cosmetic score and symptom score assessed before ablation

^b^The cosmetic score and symptom score assessed at the last follow-up visit

^c^< .05

## Discussion

Traditional treatments for symptomatic TNs include surgery and medication. Surgical options (total, subtotal thyroidectomy, lobectomy) can not only be invasive but also cause pain and cosmetic defects in patients. In addition, postsurgical long-term administration of thyroid hormone therapy is usually needed. Therefore, minimally invasive procedures such as endoscopic thyroid surgeries and levothyroxine therapy provide an alternative treatment method of TNs [21]. However, larger damage may be caused by endoscopic thyroid surgeries. The efficacy of levothyroxine is still controversial. Side effects such as reduction of bone density or atrial fibrillation may be caused by long-term use of levothyroxine [22].

In recent years, thermal ablation therapies have been introduced for treating benign TNs such as microwaves, laser and radiofrequency ablation [23, 24]. However, RFA treatment for the benign TNs is still controversial because the currently available data come mostly from limited series of patients in prospective clinical trials [17, 25–27]. Moreover, it has also been reported that thyroid nodule function may affect the efficacy of RFA treatment [13, 28]. In addition, studies have shown that thyroid nodule fluid content may affect the treatment effect of RFA [11, 29]. In fact, clinical outcomes and incidence of adverse effects are also strongly influenced by the size, type of nodules and treatment algorithm [30, 31]. In order to avoid the factors confounded the results, we previously excluded cystic nodules or TNs with less than 80% solid portion in this study. Compressive symptoms and cosmetic concerns are usually related to the volume of nodules. In this study, the TNs were categorized into small, medium, intermediate and large groups. In particular, this study evaluated the VRR of TNs, changes of cosmetic and symptom scores and post-ablation complications.

This 2 years retrospective study demonstrated that RFA was effective for treating non-functioning solid benign TNs and controlling clinical symptoms. The factor affecting treatment effect was initial volume of TNs. The VRR of the small group of nodules were larger compared to the medium, intermediate and large groups. To prevent the regrowth of nodules, we applied the moving shot technique to complete ablation of the nodules in this study. However, larger nodules required multiple RFA sessions to achieve similar VRR than smaller nodules. No nodule regrew with regrowth defined as the nodule volume increased 50% more than the previous examination. Cosmetic and pressure symptoms are significantly improved in particular intermediate and large nodules.

Regarding safety, this study showed no major complications and a low complication rate. No perinodular hematoma, nerve injury, local infection, skin burn or hypothyroidism was found, and only 12 patients (12/103, 11.6%) had a mild local pain during RFA and lasted for 1 to 3 days, which alleviated with analgesics. The low power and prolonged time of RFA in this study might result in the low complication rate.

No comparison with surgery or other treatment methods is the limitation of this retrospective study.

## Conclusions

This 2 years follow up study has shown that RFA was effective for treating non-functioning solid benign TNs with a low complication rate as well as in controlling the problems caused by TNs. The reduction rate was related to the initial volume of nodules. The best reduction rate was observed in the small group of nodules. However, multiple RFA treatments should be used in larger nodules to achieve the desired clinical outcomes. Cosmetic signs and pressure symptoms were improved significantly, particularly in the intermediate and large groups. Therefore, RFA may be used in clinical practice as a minimally invasive method for treating non-functioning solid benign TNs.

## Abbreviations

CEUS: Contrast-enhanced ultrasound
CNB: Core needle biopsy
ECG: Electrocardiogram
RFA: Radiofrequency ablation
TNs: Thyroid nodules
TSH: Thyroid stimulating hormone
VRR: Volume reduction ratio
